# Functional Stretching Exercise Submitted for Spastic Diplegic Children: A Randomized Control Study

**DOI:** 10.1155/2014/814279

**Published:** 2014-07-20

**Authors:** Mohamed Ali Elshafey, Adel Abd-Elaziem, Rana Elmarzouki Gouda

**Affiliations:** ^1^Department of Physical Therapy for Growth and Developmental Disorder in Children and Its Surgery, Faculty of Physical Therapy, Cairo University, Egypt; ^2^Faculty of Medicine, Zagazig University, Egypt; ^3^Physical Therapy Department, General Hospital of Mit Ghamr, Egypt

## Abstract

*Objective.* Studying the effect of the functional stretching exercise in diplegic children. *Design.* Children were randomly assigned into two matched groups. *Setting.* Outpatient Clinic of the Faculty of Physical Therapy, Cairo University. *Participants.* Thirty ambulant spastic diplegic children, ranging in age from five to eight years, participated in this study. *Interventions.* The control group received physical therapy program with traditional passive stretching exercises. The study group received physical therapy program with functional stretching exercises. The treatment was performed for two hours per session, three times weekly for three successive months. *Main Outcome Measure(s).* H*∖*M ratio, popliteal angle, and gait parameters were evaluated for both groups before and after treatment. *Results.* There was significant improvement in all the measuring variables for both groups in favor of study group. H*∖*M ratio was reduced, popliteal angle was increased, and gait was improved. *Conclusion(s).* Functional stretching exercises were effectively used in rehabilitation of spastic diplegic children; it reduced H*∖*M ratio, increased popliteal angle, and improved gait.

## 1. Background

Spastic children are the commonest type of cerebral palsy (CP) [1] characterized by increased resistance to passive movement due to spasticity [2]. The loss of supraspinal control over reflex arc causes spasticity [3] depending on location and severity of lesion [4, 5]. Spasticity leads to muscle contractures and bone deformities as a result of secondary structure changes of the muscles fibers [6, 7]. These changes in fiber bundles and fewer sarcomeres [8, 9] cause a decreased range of motion (ROM) [10]. Spastic diplegic children had hip flexors, hamstrings, psoas, and calf muscles tightness, leading to flexed posture [11–13].

Stretching exercises were developed to manage spasticity, including passive and active stretching, positioning, and isotonic and isokinetic stretching. The effect of stretching depends on tension applied to the soft tissue, duration, repetition in session, and daily frequency [14, 15]. Static stretching splint reduced spasticity and improved motor function [16, 17]. Stretching prevented adhesion of the joint capsule [18]. Stretching followed by passive exercise reduced hyperactive stretch reflexes [19, 20]. Slowly sustained stretch managed painful contractures [21, 22]. Prolonged muscle stretch reduced motor neuron excitability [23]. Stretching exercises increased Achilles tendon cross-sectional area, decreased gastrocnemius and soleus muscle fascicular stiffness [24], and improved walking and crouch posture [25].

On the other hand, some researches stated that stretching did not improve joint mobility in people with contractures if performed for less than seven months [26]. Prolonged muscle stretch did not efficiently treat or prevent contracture [27] and failed to provide proper treatment for spasticity [28].

A systematic review of stretching exercises for CP children reported limited evidence that manual stretching increased ROM, reduced spasticity, and improved walking because of methodological issues, small sample sizes, small number of studies, and little attention in the evaluation of active stretching in children with CP [29]. There is no evidence that stretching improved ROM during walking or other functional activities [30]. A systematic review of physical therapy treatment for CP demonstrated that stretching exercise for CP is limited because the mechanism and etiology of muscle contractures are not clear and clinical research evaluating the effectiveness of stretching techniques is inconclusive and cannot guide therapists' clinical decision making [31].

## 2. Subjects, Materials, and Methods

### 2.1. Study Design

This study was a randomized controlled trial. The study was performed over one year during the period from January 2013 to January 2014.

### 2.2. Subjects

Thirty-two ambulant spastic diplegic CP children were selected from the Outpatient Clinic of the Faculty of Physical Therapy, Cairo University. Their range of age was 5–8 years old. The experiments divided subjects into blocks according to severity of mild and moderate. Then, within each block subjects are randomly assigned to control and study groups to ensure that each treatment condition has proportion equal to control and study group. The randomized block removes dividing of the children groups as source of variability and as confounding variable (Table 1).

All children were ambulant with crouch gait pattern and had grades I and II according to gross motor function classification system and spasticity of grade 1 or grade 1^+^ according to modified Ashworth scale. All children can follow orders and have neither auditory nor visual disorders. Children were excluded if they had hip dislocation, fixed contractures or deformity, surgical intervention as surgical release, rhizotomy and tenotomy, Botulinum toxin injections, baclofen pump, osteoporosis, heart diseases, uncontrolled convulsions, and leg length discrepancy.

### 2.3. Materials

EMG Neuropac S1, Model DI 90B, SN 00030, made in Japan, Motion analysis system, Hocoma 00034, made in USA, and universal goniometry were used.

### 2.4. Methods

Each group was evaluated before and after three months of treatment by the Hoffmann reflex of soleus muscle. The soleus H-reflex is obtained by anodal stimulation of the posterior tibial nerve at the popliteal fossa. Small intensities of stimulation preferentially activate Ia fibers that excite alpha-motor neurons, thus giving rise to reflex response in the soleus H-wave that was recorded by EMG and then stimulation with higher intensities to activate axons of alpha-motor neurons and M-response was recorded. The popliteal angle was measured by the universal geometry, with the child placed in supine position and hip flexed to right angle. The stationary arm of the goniometer was placed parallel to the longitudinal axis of the femur, in line bisecting greater trochanter and the movable arm parallel to the longitudinal axis of fibula, in line bisecting lateral malleolus and the axis of the goniometer was placed over the lateral epicondyle of the femur, while the other leg in neutral extension. Stride length, stride speed, and stance phase percentages were evaluated in both groups before and after treatment by 3D motion analysis system.

## 3. Treatment Methods

The control group received the selected physical therapy program; the program included function training, balance exercise, trunk control, and passive stretching exercises for the hip flexors, hip adductors, hamstring, and calf muscle; stretching was applied for 30 sec with 30 sec rest 3–5 times for each muscle group within pain limit followed by strengthening exercise for weak muscles which was performed in three groups; each group contained ten repetitions for each weak muscle group.

**Figure 1:**
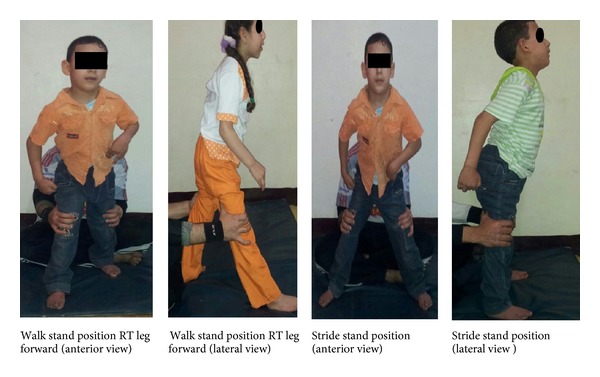
Illustration of walk stand and stride stand positions.

The study group received the same selected physical therapy program also; however, stretching exercise and function training were performed together. The functional stretching exercises were performed by training the child to maintain walk standing and stride standing positions with gradual increase of distance between lower limbs with extended knees to stretch lower limb muscle, and the child was trained to stoop and recover from these positions. The child was trained to walk with full extended knees and hips in slight abduction with correction of lower limb rotation. Strengthening of abdominal and back muscles was performed with hip joint abductions and knee joint extension. The program was performed gradually according to child tolerance within pain limit also and rest given when required (Figure 1).

### 3.1. Functional Stretching Exercise Program

The child was trained to be maintained standing in walk stand position and gradually increase distance between both legs to stretch lower extremities muscles with RT leg forward then with LT leg forward.

The child was trained to be maintained standing in stride standing position and gradually increase distance between legs to stretch tight hip adductors muscles of both sides.

The child was trained to stand on RT leg in complete extension while holding the other leg in abduction to stretch hip adductor of the raised leg and then reverse the leg position.

The child was trained to walk while manually holding knee joint in full extension and hip joint in slight abduction to stretch all lower limb muscles with correction of lower extremities rotation.

Stoop and recovery were performed from walk and stride stand position and gradually increased distance between both legs according to the child tolerance.

Strengthening of lower back extensor and hip extensors was performed prone on wedge and both legs abducted and knees extended; also strengthening of abdominal muscles was performed supine on mat with legs abducted and knees extended.

Balance exercise was performed also from walk stand and stride stand positions.

Both groups wear ankle foot orthosis during day time and knee ankle foot orthosis at bed time. Both groups received the treatment program for two hours three times weekly for three successive months.

### 3.2. Data Analysis

The statistical analyses were performed with the aid of the statistical package of social sciences (SPSS) version 20. Descriptive statistics (mean and standard deviation) were computed for all data. The paired *t*-test was applied for comparison within the group and the independent *t*-test was applied to compare between both groups before and after treatment. Cronbach's alpha applied for measuring internal consistency, the statistical power, and minimal clinical difference depending on the Anchor method were calculated.

## 4. Result

There was no significant difference between both groups in age (*P* > 0.05); the mean age was 6.23 ± 0.942 and 6.06 ± 0.951 for control and study group, respectively. There was also no significant difference between both groups in gender, spasticity, and gross motor function classification system level distributed between both groups; the Chi-squared value was 0.67, 0.8, and 0.7, respectively (*P* > 0.05).

Comparison between right and left leg in both groups revealed that there was no significant difference between both sides (*P* > 0.05) as illustrated in Table 2. The internal consistency was expressed as excellent validity according to the values of Cronbach's alpha for the different measured variables according to Table 3.

**Figure 2:**
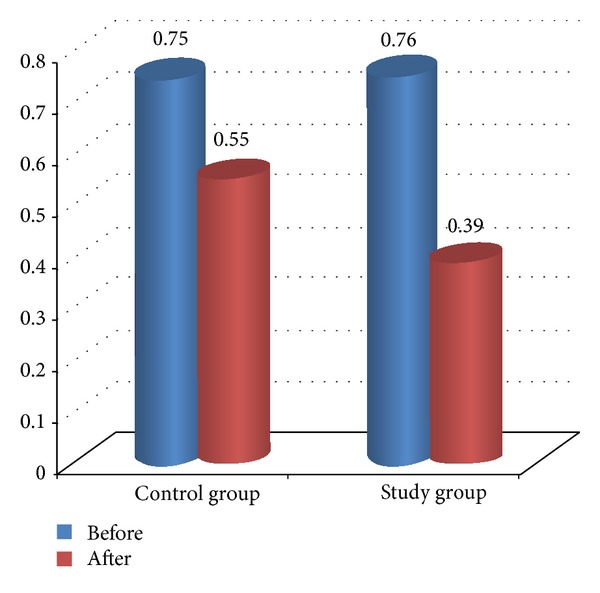
Comparison between pre- and posttreatment in H*∖*M ratio for both groups.

**Figure 3:**
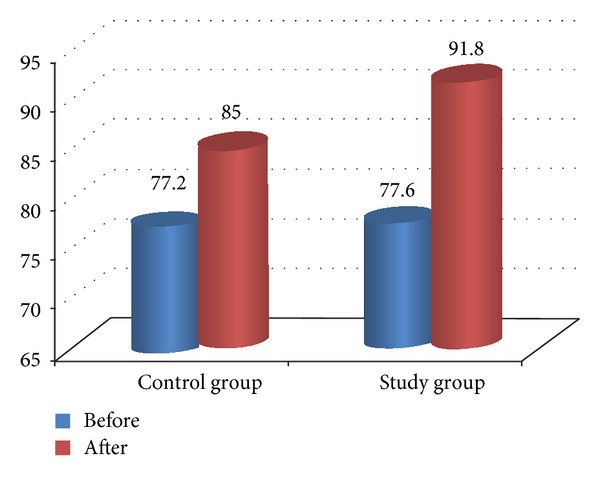
Comparison between pre- and posttreatment in popliteal angle for both groups.

**Figure 4:**
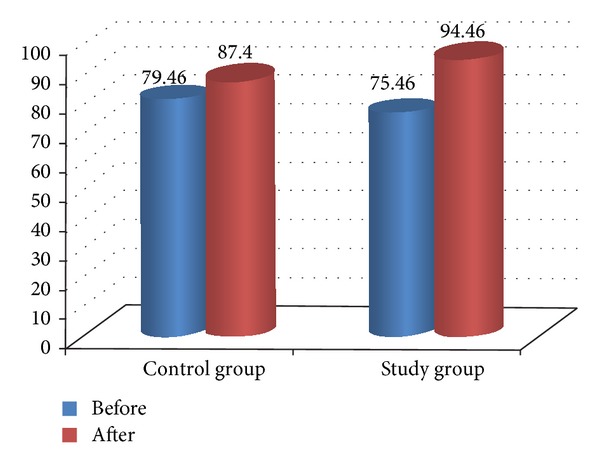
Comparison between pre- and posttreatment in stride length for both groups.

**Figure 5:**
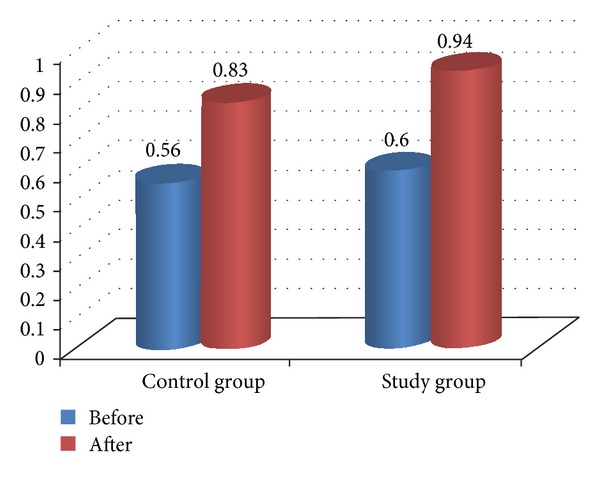
Comparison between pre- and posttreatment in stride speed for both groups.

**Figure 6:**
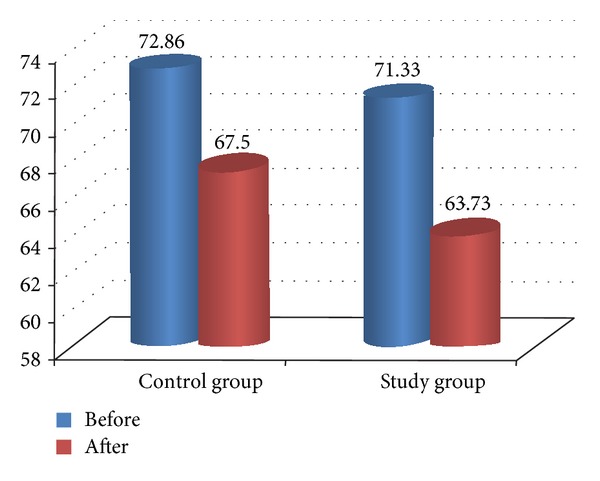
Comparison between pre- and posttreatment in stance phase % for both groups.

Comparison between pre- and posttreatment for both groups revealed that there was significant improvement in both groups after treatment in all measured variables (*P* < 0.05) as illustrated in Table 4 and Figures 2, 3, 4, 5, and 6. The posttreatment comparison revealed that there was significant improvement in favor of the study group (*P* < 0.05) as illustrated in Table 4.

The statistical power values showed greater effect for both groups in favor of the study group. The minimal clinical difference was ≤ 0.5 SD representing a moderate effect size and this is corresponding to the minimal important difference (Table 5).

## 5. Discussion

Spastic diplegic CP children suffered from muscular tightness and loss of flexibility, leading to mechanical problems, loss of range of motion, and limited executive function abilities. Stretching exercise was applied to increase soft tissue flexibility in CP children;some researchers reported that regular stretching does not produce clinical changes in joint mobility, spasticity, and function activities [32].

The functional stretching exercises were designed to treat soft tissue flexibility problems during function training; stretching is applied in unique way depending on the concepts of overcorrection of deformities and prolonged stretching to utilize the inhibitory effect of stretching in improving function training during physical therapy treatment in order to optimize motor performance. Different techniques were used for spasticity evaluation but we used H-reflex because it is standardized procedure and had greater evidence of validity [33]. The popliteal angle was measured because it is a strong clinical indicator for hamstring muscle tightness and needed for surgical intervention. Stride length, stride speed, and stance phase percentages were measured by 3D gait analysis as indicator of program effect on gait parameters.

The result of the study demonstrated that there was no significant difference between and within the groups before treatment and the internal consistency expressed excellent validity. There was significant improvement in all measured variables for both groups in favor of functional stretching program as the child was functionally trained with correct movement pattern and stretching performed during the whole session, as stretching effect on spasticity lasts for one or two minutes. The result of popliteal angle showed significant improvement after treatment after application of the functional stretching program so it efficiently solved the mechanical problems of the knee joint as hamstring tightness is the main cause of crouch posture.

Walk stand position stretched mainly hamstring muscle in the forward leg and hip flexors and calf muscle of the behind leg, while stride stand position stretched hip adductors muscles. Standing and walking were applied in normal functional pattern that provides a strong proprioceptive stimulation leading to correct body image and Ingram.

The function training exercise was performed with complete knee joint and hip joint abduction and ankle in right angle. Antispastic position applied on hips was abducted at nearly a 45° angle and externally rotated, and the knees were extended and ankles right angle [34]. Antispastic positions prevented muscle contractures and joint limitation in spastic diplegia children [35]. Active movement application during passive stretching improved motor control performance and functional capability [36].

Integration of sensory stimulation during teaching the child function skills with correction of abnormal motor pattern enhanced learning process and acquisition of motor skills, leading to faster transferring of learned skill from the cognitive stage to the autonomous stage which in turn leads to perfect performance of skill and function recovery as motor learning is a set of internal processes associated with practice or experience leading to relatively permanent changes in executive functions and behaviors.

*Limitation.* A limited number of children participated in the study.

*Recommendation.* Additional research is recommended to determine the long lasting effect of functional stretching exercises on spasticity and function activities for spastic diplegic children.

## 6. Conclusion

Functional stretching exercises are effective methods used in rehabilitation of spastic diplegic children; it reduced H*∖*M ratio, increased popliteal angle, and improved gait.

## Figures and Tables

**Table 1:** Description of block randomization.

Severity according to gross motor function classification system	Treatment	
	Control	Study
Grade I	8	8
Grade II	8	8

**Table 2:** Pretreatment comparison between right and left sides in each group.

Variables	Control group		*P* value	Study group		*P* value
	RT $\overset{-}{X}\pm \text{SD}$	LT $\overset{-}{X}\pm \text{SD}$		RT $\overset{-}{X}\pm \text{SD}$	LT $\overset{-}{X}\pm \text{SD}$	
H*∖*M ratio	0.75 ± 0.09	0.77 ± 0.07	0.348∗	0.76 ± 0.07	0.75 ± 0.01	0.792∗
Popliteal angle	77.2 ± 5.58	76.93 ± 5.63	0.77∗	7.6 ± 7.32	76.3 ± 7.56	0.313∗
Stride length	79.46 ± 7.8	81.13 ± 5.82	0.16∗	75.46 ± 19.5	80.53 ± 6.0	0.269∗
Stride speed	0.56 ± 0.09	0.59 ± 0.6	0.234∗	0.6 ± 0.11	0.59 ± 0.085	0.539∗
Stance phase %	72.86 ± 3.7	71.66 ± 3.19	0.212∗	71.33 ± 4.16	70.6 ± 4	0.469∗

$\overset{-}{X}\pm \text{SD}$: mean ± standard deviation; *P*: level of significant; ∗nonsignificant.

**Table 3:** The internal consistency of the different measured variables.

Variable	Cronbach's alpha
H*∖*M ratio	0.97∗
Stride length	0.96∗
Stride speed	0.96∗
Stance phase	0.95∗
Popliteal angle	0.94∗

*Excellent validity.

**Table 4:** Comparison between both groups in all measured variable.

Variable	Time	Control group	Study group	*P* value	Standard error
		$\overset{-}{X}\pm \text{SD}$	$\overset{-}{X}\pm \text{SD}$		
H*∖*M ratio	Before	0.75 ± 0.09	0.76 ± 0.07	0.66∗∗	0.28
	After	0.55 ± 0.04	0.39 ± 00.7	0.001∗	0.27
	*P* value	0.001∗	0.001∗		
	Standard error	0.192	0.14		

Popliteal angle	Before	77.2 ± 5.58	77.6 ± 7.32	0.868∗∗	1.53
	After	85 ± 5.71	91.8 ± 5.7	0.003∗	1.36
	*P* value	0.001∗	0.001∗		
	Standard error	0.3	0.63		

Stride length	Before	79.46 ± 7.8	75.46 ± 19.5	0.468∗∗	1.77
	After	87.4 ± 7.45	94.46 ± 3.87	0.003∗	1.61
	*P* value	0.001∗	0.001∗		
	Standard error	0.3	0.98		

Stride speed	Before	0.56 ± 0.09	0.6 ± 0.11	0.325∗∗	0.03
	After	0.836 ± 0.89	0.94 ± 0.038	0.001∗	0.01
	*P* value	0.001∗	0.001∗		
	Standard error	0.27	0.26		

Stance phase %	Before	72.86 ± 3.7	71.33 ± 4.16	0.296∗∗	1.45
	After	67.5 ± 3.79	63.73 ± 1.48	0.001∗	1.14
	*P* value	0.001∗	0.001∗		
	Standard error	1.54	1.19		

$\overset{-}{X}\pm \text{SD}$: mean ± standard deviation; *P*: level of significant; *significant; **nonsignificant.

**Table 5:** Statistical power and minimal clinical difference for both groups.

Variables	Statistical power (%)		Minimal clinical difference	
	Control group	Study group	Control group	Study group
H*∖*M ratio	83∗	98∗	0.02	0.015
Popliteal angle	80∗	86.9∗	2.5	2.7
Stride length	83∗	94∗	3.2	1.8
Stride speed	80∗	86∗	0.04	0.012
Stance phase	87∗	93∗	1.5	0.5

*Statistical power ≥ 80% has large effect.

## Abbreviations

3D: Three dimensions
CP: Cerebral palsy
EMG: Electromyography
H-reflex: The Hoffmann reflex
ROM: Range of motion
SPSS: Statistical package of social sciences.
