# Corrigendum to “Functional Stretching Exercise Submitted for Spastic Diplegic Children: A Randomized Control Study”

**DOI:** 10.1155/2016/1615024

**Published:** 2016-09-18

**Authors:** Mohamed Ali Elshafey, Adel Abd-Elaziem, Rana Elmarzouki Gouda

**Affiliations:** ^1^Department of Physical Therapy for Growth and Developmental Disorder in Children and Its Surgery, Faculty of Physical Therapy, Cairo University, Egypt; ^2^Faculty of Medicine, Zagazig University, Egypt; ^3^Physical Therapy Department, General Hospital of Mit Ghamr, Egypt

In the article titled “Functional Stretching Exercise Submitted for Spastic Diplegic Children: A Randomized Control Study” [1], there was an error in Table  2, which should be corrected as follows: 7.6 ± 7.32 should be 76 ± 7.32. The revised table is below.

## Figures and Tables

**Table 2:** Pretreatment comparison between right and left side in each group.

Variables	Control group		*P* value	Study group		*P* value
	RT $\overset{-}{X}\pm SD$	LT $\overset{-}{X}\pm SD$		RT $\overset{-}{X}\pm SD$	LT $\overset{-}{X}\pm SD$	
H∖M ratio	0.75 ± 0.09	0.77 ± 0.07	0.348^**∗**^	0.76 ± 0.07	0.75 ± 0.01	0.792^**∗**^
Popliteal angle	77.2 ± 5.58	76.93 ± 5.63	0.77^**∗**^	76 ± 7.32	76.3 ± 7.56	0.313^**∗**^
Stride length	79.46 ± 7.8	81.13 ± 5.82	0.16^**∗**^	75.46 ± 19.5	80.53 ± 6.0	0.269^**∗**^
Stride speed	0.56 ± 0.09	0.59 ± 0.6	0.234^**∗**^	0.6 ± 0.11	0.59 ± 0.085	0.539^**∗**^
Stance phase %	72.86 ± 3.7	71.66 ± 3.19	0.212^**∗**^	71.33 ± 4.16	70.6 ± 4	0.469^**∗**^

$\overset{-}{X}\pm SD$: mean ± standard deviation; *P*: level of significance; ^**∗**^nonsignificant.

## References

[1] Elshafey M. A., Abd-Elaziem A., Gouda R. E. (2014). Functional stretching exercise submitted for spastic diplegic children: A Randomized Control Study. *Rehabilitation Research and Practice*.

